# Monitoring Cd^2+^ in oily wastewater using an aptamer-graphene field-effect transistor with a selective wetting surface†

**DOI:** 10.1039/d2na00416j

**Published:** 2022-12-13

**Authors:** Hao Wang, Zhuang Hao, Cong Huang, Feiran Li, Yunlu Pan

**Affiliations:** a Key Laboratory of Micro-Systems and Micro-Structures Manufacturing of Ministry of Education, Harbin Institute of Technology Harbin 150001 Heilongjiang China yunlupan@hit.edu.cn lifeiran@hit.edu.cn; b School of Mechatronics Engineering, Harbin Institute of Technology Harbin 150001 Heilongjiang China

## Abstract

The discharge of oily industrial wastewater containing heavy metal ions with the development of industry severely threatens the environment and human health. Therefore, it is of great significance to monitor the concentration of heavy metal ions in oily wastewater quickly and effectively. Here, an integrated Cd^2+^ monitoring system consisting of an aptamer-graphene field-effect transistor (A-GFET), oleophobic/hydrophilic surface and monitoring-alarm circuits was presented for monitoring the Cd^2+^ concentration in oily wastewater. In the system, oil and other impurities in wastewater are isolated by an oleophobic/hydrophilic membrane before detection. The concentration of Cd^2+^ is then detected by a graphene field-effect transistor with a Cd^2+^ aptamer modifying the graphene channel. Finally, the detected signal is collected and processed by signal processing circuits to judge whether the Cd^2+^ concentration exceeds the standard. Experimental results demonstrated that the separation efficiency of the oleophobic/hydrophilic membrane to an oil/water mixture was up to 99.9%, exhibiting a high oil/water separation ability. The A-GFET detecting platform could respond to changes in the Cd^2+^ concentration within 10 min with a limit of detection (LOD) of 0.125 pM. The sensitivity of this detection platform to Cd^2+^ near 1 nM was 7.643 × 10^−2^ nM^−1^. Compared with control ions (Cr^3+^, Pb^2+^, Mg^2+^, Fe^3+^), this detection platform exhibited a high specificity to Cd^2+^. Moreover, the system could send out a photoacoustic alarm signal when the Cd^2+^ concentration in the monitoring solution exceeds the preset value. Therefore, the system is practical for monitoring the concentration of heavy metal ions in oily wastewater.

## Abstract 2

An integrated system realizes the direct detection of Cd^2+^ in oily wastewater without sample collection and pretreatment.

## Graphical abstract

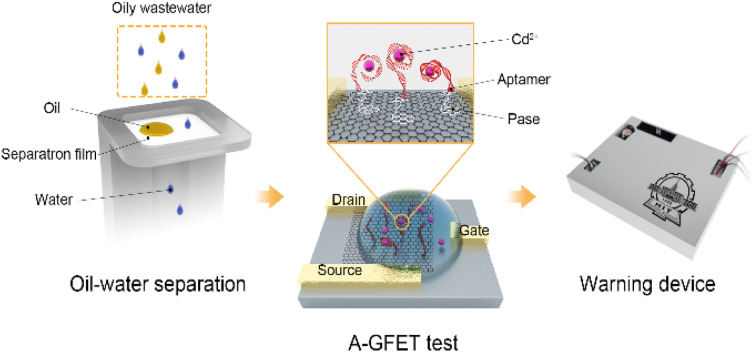

## 1 Introduction

With the development of industry, the pollution of heavy metals in the water environment is becoming increasingly severe due to the discharge of oily industrial wastewater containing heavy metal ions.^1,2^ Some adverse events caused by extreme heavy metals in the water environment have been reported, seriously threatening environmental safety and human health. Heavy metals, such as lead, cadmium, and mercury, can cause serious harm to biological systems, such as the nervous, respiratory, and cardiovascular systems, when the concentration exceeds normal values.^3–7^ Therefore, it would be of great significance to monitor the concentration of heavy metal ions quickly and effectively. In the integrated wastewater discharge standard issued by the State Environmental Protection Administration of China, the maximum allowable emission concentrations of heavy metals in wastewater are specified,^8^ and the emission standards of some heavy metals are shown in Table S1.† Traditional detection methods for heavy metal ions mainly include atomic absorption spectrometry (AAS), mass spectrometry, and X-ray fluorescence spectrometry.^9–12^ These traditional methods generally require overnight pretreatment of the samples. Additionally, the relatively long detection cycle and large bulk of the detection equipment mean that the conventional methods cannot meet the needs for rapid, effective, and on-site detection. Portable devices that could achieve the rapid and effective detection of metal ions could play an important role in preventing heavy metal pollution in oily industrial wastewater.

Graphene, a two-dimensional material with excellent electrical properties, has attracted considerable attention since it was first prepared in 2004.^13^ Owing to its high sensitivity to its surface charge distributions, a graphene-based field-effect transistor can convert changes in the charge distribution on the graphene surface into detectable electrical signals.^14,15^ As a new identification element obtained by systematic evolution of ligands by exponential enrichment (SELEX) technology, aptamers have the advantage of being easy to synthesize and having good stability compared with antibodies, enzymes, and other biological probes.^16–18^ Aptamer-graphene field-effect transistors (A-GFETs), in which the aptamer is modified on the surface of the graphene channel for use as a biological probe, have been successfully applied in the detection of DNA,^19^ proteins,^20,21^ and other organic compounds.^22^ Moreover, several studies using A-GFETs for ion detection have also been reported, with the transistors exhibiting high specificity, fast response, and low limit of detection compared with traditional detection methods.^23–25^ The A-GFET sensor can be fabricated on a tiny silicon chip (2 × 2 cm); thereby promoting the development of miniaturization and portability of the ion-detection equipment. Nevertheless, some problems remain to be solved when the A-GFET is applied for the detection of metal ions in oily wastewater.

Currently, most studies of A-GFETs have been conducted using artificial samples with a relatively simple composition since the surface integrity and electrical properties of graphene are highly susceptible to other substances in the detecting environment.^21,26^ In particular, the difficulty of achieving oil/water separation further limits the application of A-GFETs for detecting heavy metal ions in oily industrial wastewater. Hence, the antifouling treatment of graphene surface is a critical challenge in applying A-GFET biosensors to actual samples. In recent years, despite selectively wetted surfaces having attracted considerable attention due to their high separation efficiency for oil/water separation,^27–30^ the problem of oil deposition on the graphene surface has still not been effectively solved.

In this study, an integrated monitoring system consisting of an aptamer-graphene field-effect transistor, oleophobic/hydrophilic surfaces, and monitoring-alarm circuits is presented. Benefiting from the effective filtration of the oleophobic/hydrophilic membrane for oil, the integrated system could realize the direct detection of Cd^2+^ in oily wastewater. In this system, the prepared oleophobic/hydrophilic membrane could effectively achieve the filtration of oil pollution in wastewater, thus allowing solving the problem of oil deposition on graphene surfaces in practical applications. At the same time, the integrated circuit board could realize the timely collection and processing of signals, meeting the needs for on-site detection. Also, the application of such A-GFET detection technology in Cd^2+^ detection could effectively shorten the required time for performing the detection process. In addition, the integrated monitoring system prepared in this study is more portable compared with the conventional ion-detection equipment owing to the small size of the system components.

**Fig. 1:**
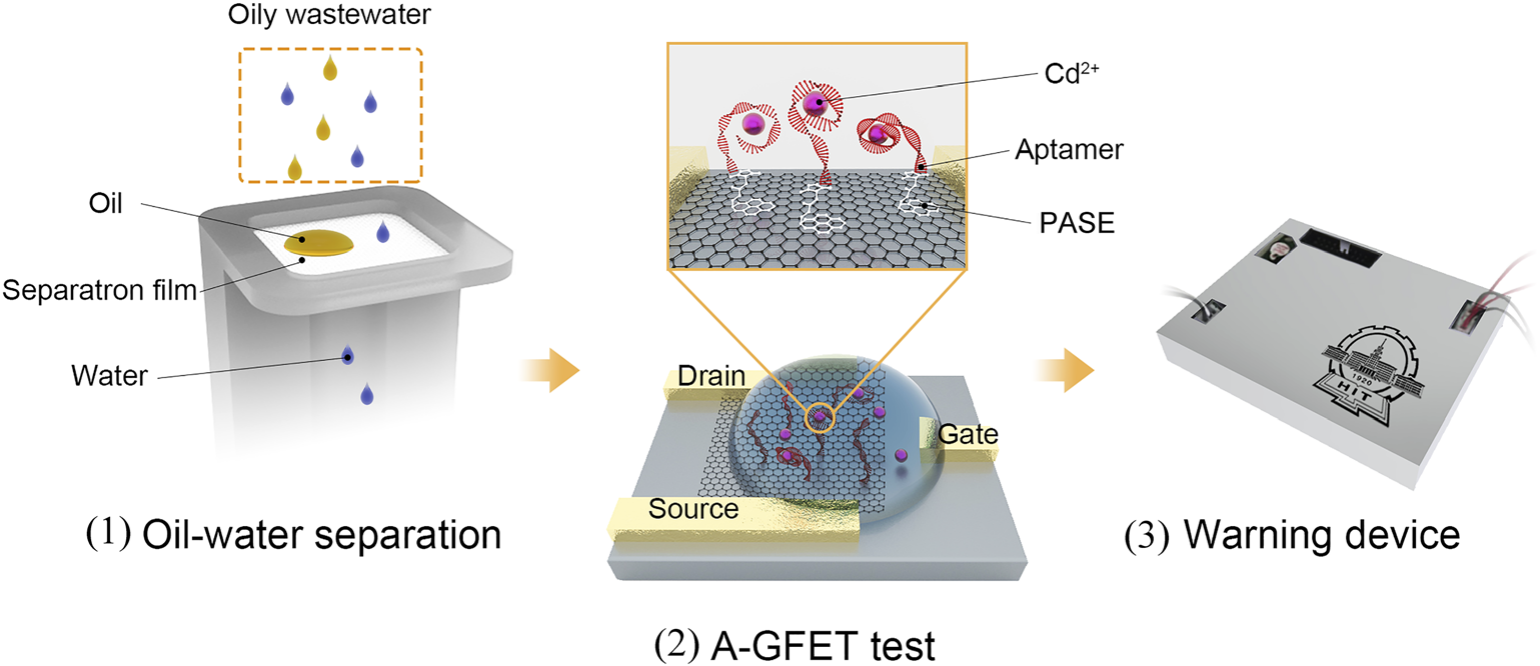
Schematic diagram of the Cd^2+^-detection process in oily wastewater. The detection can be divided into three processes: (1) oil–water separation, (2) Cd^2+^ detection using the A-GFET detecting platform, (3) analysis of the data and generation of an alarm signal by the PCB board.

The Cd^2+^-detection process in oily wastewater is shown in Fig. 1. The working process of the system can be divided into three parts. First, the oleophobic/hydrophilic membrane was prepared using fluorosurfactant FS-50, TiO_2_ nanoparticles, and stainless-steel mesh to stop the oil and other solid pollutants from contacting the graphene channel. Second, the A-GFET detecting platform was prepared to achieve the detection of Cd^2+^. In this setup, the solution, after filtration, is in contact with the graphene channel, and the Cd^2+^ in the solution is captured by aptamers modified on the surface of the graphene simultaneously. The aptamers transform from an extended long-chain structure into a compact and stable structure after combining with Cd^2+^, which can pull more negative charges produced by the hydrolysis of phosphate groups in aptamers close to the surface of the graphene, changing the surface charge distribution of graphene. As a result, the electrical signal of the graphene-based field-effect transistor changes accordingly. Finally, the detected signal is collected and processed by the monitoring-alarm circuits, which allows then judging whether the Cd^2+^ concentration exceeds the standard. The integrated monitoring system is a practical solution for monitoring the concentration of heavy metal ions in oily wastewater.

The experimental results demonstrated that the A-GFET detecting platform could respond to changes in Cd^2+^ concentration within 10 min with high specificity and a low LOD of 0.125 pM. Also, the oleophobic/hydrophilic membrane could effectively eliminate the influence of oil pollution on the detection performance of the A-GFET detection platform for Cd^2+^, achieving a high separation efficiency of over 99.9% for oil/water mixtures.

## 2 Experimental and methods

### 2.1 Materials

Chemical vapor deposition (CVD) graphene was purchased from Graphenea (Cambridge, MA), while 285 nm SiO_2_/Si was ordered from UniversityWafer Inc. (Boston, MA, USA). 1-Pyrenebutanoic acid succinimidyl ester (PASE), dimethylformamide (DMF), and ethanolamine were purchased from Sigma-Aldrich (St. Louis, MO, USA). Cadmium chloride hemi(pentahydrate) was purchased from Aladdin (Shanghai, China). The Cd^2+^ aptamer (sequence 5′-NH_2_- CTC AGG ACG GGT TCA CAG TCC GTT GTC -3′) was synthesized and purified by Sangong Biotech (Shanghai, China). Fluorosurfactant FS-50 was purchased from Dupont (Delaware, MA, USA). Spray-Mount Super 75 adhesive was purchased from 3M (Shenzhen, China). Titanium oxide (TiO_2_) anatase (100 nm) was purchased from Macklin (Shanghai, China). The main agent and curing agent of 184 silicone rubber (PDMS) and AB adhesive were purchased from Yihui Adhesive (Guangdong, China).

### 2.2 A-GFET detecting platform fabrication

The fabrication processes for the gold electrodes on the surface of a silicon wafer covered with a layer of SiO_2_ (285 nm) are illustrated in Fig. S1.† After etching the copper substrate, CVD graphene was transferred between gold electrodes.

PASE was used as a linker to modify the aptamer onto the graphene surface. The silicon wafer was first immersed in 5 mM PASE solution at room temperature for 3 h and then rinsed with DMF to remove free PASE not fixed to graphene. Subsequently, a droplet of aptamer solution (50 μl) with a concentration of 100 nM was dropped onto the graphene overnight at room temperature. Finally, the A-GFET detecting platform was immersed in 100 mM ethanolamine for 1 h to inactivate the reactive groups in the PASE molecules that were not bound to the aptamer, and then rinsed off with ethanol and deionized water successively.

The pyrenyl group at one end of the PASE molecule was combined with graphene by π–π stacking, while the ester group at the other end was combined with the amino group on the aptamer by Schiff base condensation to achieve modification of the aptamer on the graphene surface. The principles for the graphene surface modification and Cd^2+^ detection are shown in Fig. S2 and S3.†

### 2.3 Oleophobic/hydrophilic membrane fabrication

To fabricate the oleophobic/hydrophilic membrane, 3 g of fluorosurfactant (FS-50) was added to 50 mL of ethanol. The mixture was then stirred at room temperature for 20 min. Then 1.2 g of TiO_2_ nanoparticles was added to the FS-50/ethanol solution. The mixture was stirred magnetically at room temperature for another 1 h to form a suspension. Subsequently, the suspension was evenly sprayed on the surface of the stainless-steel mesh after cleaning with alcohol. A layer of 3M adhesive was sprayed on the surface of the stainless-steel mesh before spraying the suspension, which could allow the nanoparticles to adhere to the surface of the stainless-steel mesh tightly. Finally, the stainless-steel mesh was put into a drying box at 80 °C for 30 min to evaporate the ethanol.^28^ The physical model of the oleophobic/hydrophilic membrane is shown in Fig. S4†

### 2.4 Monitoring-alarm circuit design

The system included a power supply, voltage output, signal acquisition, signal processing, and photoacoustic alarm circuits. In the power-supply circuit, the voltage of 5 V was supplied directly by the transformer, while a voltage of 2.5 V was outputted by the REF5025 while voltages of 1.8 and 3.3 V were outputted by the AMS1117 power-supply modules. The voltage output circuit included a drain-source voltage (*V*_ds_) output and gate voltage output (*V*_g_). A *V*_ds_ of 10 mV was supplied by a voltage regulator circuit, and the *V*_g_ was supplied by DAC8831IBD, ranging from 0 to 0.5 V in steps of 1 mV. During the detection process, the drain-source current (*I*_ds_) of the A-GFET detecting platform was converted into a voltage signal by an amplifier circuit and then collected by the A/D converters ADS1274 and THS4524. The collected signal was processed by the microcontroller STM32F429IGT6, and the system would send out a photoacoustic alarm signal if certain preset conditions were met.

### 2.5 Cd^2+^-monitoring system fabrication

The main agent and curing agent of PDMS were mixed with a ratio of 10 : 1 in a plastic Petri dish with adequate stirred. To remove the bubbles created during the mixing process, the mixture was put into a vacuum box and allowed to stand for 24 h. Subsequently, the mixture was poured into a mold made by 3D printing and placed in a drying box at 120 °C for 1 h to make the PDMS solidify. The oleophobic/hydrophilic membrane was cut into a 2 × 2 cm square piece and then fixed to the solidified PDMS with AB adhesive. The drain, source, and gate electrode on the A-GFET detecting platform were led out by wires using silver conductive adhesive before fixing to the PDMS. The lead-out wires were connected to the detection platform interface on the PCB board for achieving the signal acquisition for the A-GFET detection platform. The housing of the Cd^2+^-monitoring system was made by 3D printing.

## 3 Results and discussion

### 3.1 Characterization of the surface modification on graphene

**Fig. 2:**
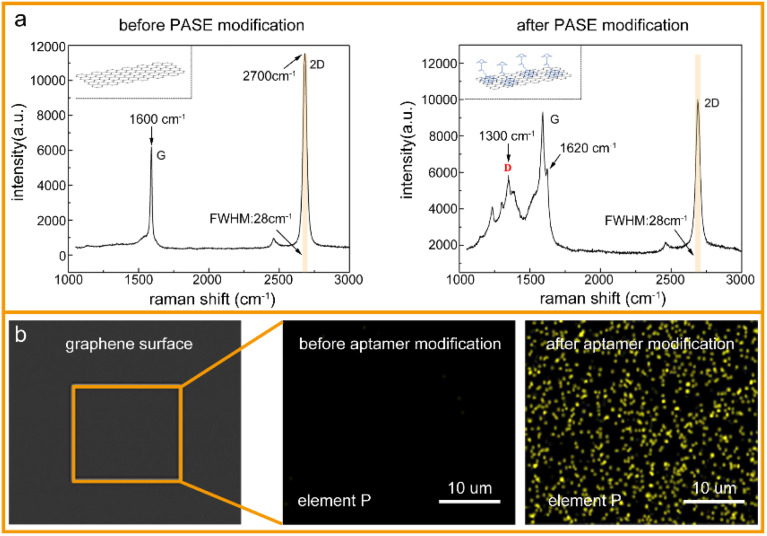
Surface modification of graphene: (a) Raman spectroscopic characterization of graphene before and after PASE modification, (b) EDS image of P element changes on the surface of graphene before and after aptamer modification.

To verify the successful modification of the PASE and aptamer, the graphene surface was characterized after each functionalization by Raman spectroscopy and energy spectrometry, respectively. As shown in Fig. 2a, the Raman spectrum of monolayer graphene usually includes two characteristic peaks: a G-peak (1600 cm^−1^) and 2D-peak (2700 cm^−1^). After PASE modification, a D-peak appeared at 1300 cm^−1^, and G-peak splitting at 1620 cm^−1^, where the peak ratio of the 2D-peak to G-peak in the Raman spectrum of graphene decreased from 1.91 to 1.04, which could be due to the binding of graphene and the pyrene groups on PASE. In addition, the full width at half maximum (FWHM) of the 2D-peak was always 28 cm^−1^, both before and after PASE modification, which could be evidence of the monolayer graphene. That is, the modification of PASE was successful. Compared with the PASE molecule in the chemical element composition, the aptamer (single-stranded DNA) had phosphorus. Energy dispersive spectroscopy (EDS) showed that the phosphorus on the graphene surface was significantly increased after aptamer modification (Fig. 2b). Considering these experimental results, it could be concluded that the aptamer was successfully modified on the graphene using PASE as an intermedium.

### 3.2 Detection of Cd^2+^ in aqueous solutions by A-GFET

**Fig. 3:**
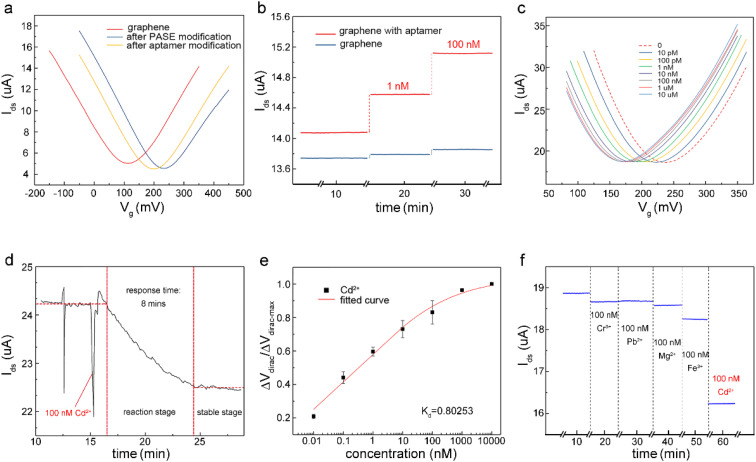
(a) Electrical characterization of graphene, graphene after modifying with PASE, and graphene after modifying with the aptamer (*V*_ds_: 10 mV). (b) Response of bare graphene and graphene after modifying the aptamer in Cd^2+^ solution at a certain concentration, showing that the effect of the change in the Cd^2+^ concentration in solution on the bare graphene could be ignored (*V*_gs_: 350 mV, *V*_ds_: 10 mV). (c) Transfer characteristic curves of graphene after modification with aptamers measured while the detection platform was exposed to Cd^2+^ solutions ranging from 10 pM to 10 μM (*V*_ds_: 10 mV). (d) Response time of the detection platform for Cd^2+^, which was about 8 min (*V*_gs_: 50 mV, *V*_ds_: 10 mV). (e) Normalized Dirac point shift Δ*V*_dirac_/Δ*V*_dirac-max_ as a function of Cd^2+^ concentrations. (f) Comparison with control ions (Cr^3+^, Pb^2+^, Mg^2+^, Fe^3+^), whereby the detection platform showed a larger response to Cd^2+^ (*V*_gs_: 50 mV, *V*_ds_: 10 mV). The above experiments were conducted in aqueous solutions.

The detection capability of the A-GFET was investigated with exposure of the graphene channel to Cd^2+^ in aqueous solutions. Before detecting Cd^2+^, transfer characteristic curves of graphene were measured after PASE and aptamer modification to explore the doping effect of the PASE and aptamer on graphene (Fig. 3a). After PASE modification, the voltage at the Dirac point (the lowest point of the graphene transfer characteristic curve) increased from 115 mV to 230 mV, indicating that p-type doping of graphene was generated after the modification of PASE. Inversely, the voltage at the Dirac point decreased from 230 mV to 200 mV after aptamer modification, implying n-type doping of graphene was generated. These changes also indicated the successful modification of the PASE and aptamer. We discuss these changes in the ESI.† To verify that graphene did not respond to changes in the Cd^2+^ concentration before modifying the aptamer, signal response (*I*_ds_) of graphene before and after aptamer modification to solutions with Cd^2+^ concentration of 1 nM and 100 nM were measured respectively (Fig. 3b). It was found that *I*_ds_ increased from 14.08 μA to 14.57 μA and 15.12 μA when the graphene with aptamer was exposed to and left in Cd^2+^ solutions for 10 min with concentrations of 1 nM and 100 nM, respectively. By contrast, *I*_ds_ changed very little when bare graphene was treated uniformly. To investigate the detection capability of the A-GFET detection platform for Cd^2+^, transfer characteristic curves of graphene with aptamer exposure to Cd^2+^ in aqueous solutions ranging from 10 pM to 10 μM were measured (Fig. 3c). As the concentration of Cd^2+^ increased, the transfer characteristic curve of graphene shifted consistently to the negative direction of the *x*-axis. Also, the gate voltage of the Dirac point gradually decreased from 230 mV to 175 mV. Before detection, the graphene channel was kept in each concentration for 10 min to ensure the aptamer could bind to Cd^2+^ adequately.

To investigate the response time of the detection platform to Cd^2+^, we monitored the change of *I*_ds_ with exposure of the detection platform to 100 nM Cd^2+^ solution after signal stabilization in solution without Cd^2+^. As shown in Fig. 3d, the detection process of the platform could be divided into two stages: the reaction stage and stable stage. The time required in the reaction stage could be regarded as the response time of the detection platform (about 8 min). In addition, the experimental data were normalized in order to eliminate the signal deviation caused by the difference between the detection platforms (Fig. 3e). The normalized data could be fitted by eqn (1):^31^1
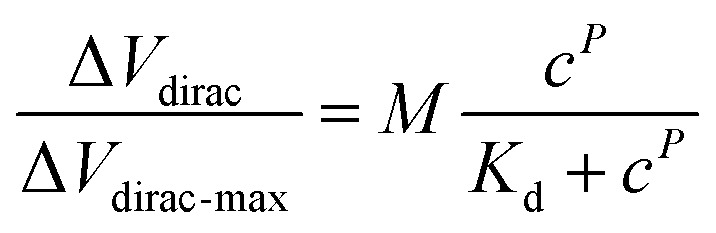
where *M* is the compensation factor, *c* is the concentration of Cd^2+^ in the solution, *P* is the synergistic factor, *K*_d_ is the constant of the dissociation equilibrium. In this experiment, *K*_d_ was 0.8 nM and the limit of detection (LOD) of the detection platform for Cd^2+^ was 0.125 pM (more information about the LOD can be found in the ESI†). As for the sensitivity of the detection platform, we used the slope of the fitting curve shown in Fig. 3e at a certain concentration to express the sensor's sensitivity near this concentration, and the sensitivity of this sensor to Cd^2+^ near 1 nM was calculated as 7.643 × 10^−2^ nM^−1^. Comparison of some important characteristics of the proposed detection method for Cd^2+^ with some previously reported studies is shown in Table S2.† It could be found that the A-GFET detecting platform proposed in this study was comparable or superior to other reported studies for Cd^2+^ detection with a lower LOD and a relatively short response time. Furthermore, to investigate the specificity of the detection platform to Cd^2+^, the signal response of the platform to control ions (Cr^3+^, Pb^2+^, Mg^2+^, and Fe^3+^) with a concentration of 100 nM was measured (Fig. 3f). Compared with Cd^2+^, for which *I*_ds_ decreased significantly, there was only a relatively small change in the drain-source current response compared to the other control ions. It could be concluded from these experimental results that the detection platform could respond to changes in the Cd^2+^ concentrations with a LOD of 0.125 pM after modifying aptamer on the surface of the graphene channel. Also, compared with control ions, such as Cr^3+^, Pb^2+^, Mg^2+^, and Fe^3+^, the detection platform had a high specificity to Cd^2+^ (IV transfer characteristic curves of the response signal of the GFET detecting platform to Cr^3+^, Pb^2+^, Mg^2+^, Fe^3+^, and Cd^2+^ are shown in Fig. S9†).

### 3.3 Alarm experiments

**Fig. 4:**
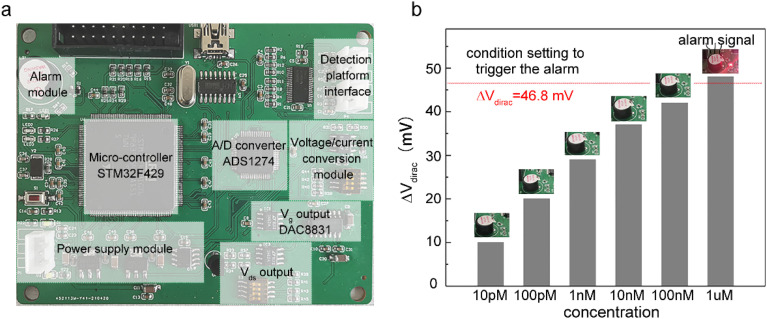
(a) Circuit board used for collecting, analyzing, processing data, and sending an alarm signal. (b) The circuit part will send out a photoacoustic alarm signal when the concentration of Cd^2+^ in the tested solution rises to a certain concentration.

Alarm experiments were carried out to verify the function of the circuits. As shown in Fig. 4a, the monitoring-alarm circuits included a microcontroller (STM32F429), A/D converter (ADS1274), *V*_g_ output (DAC8831), *V*_ds_ output, alarm module (buzzer and LED light), power-supply module (REF5025 and AMS1117), voltage/current conversion module (for converting *I*_ds_ into a voltage signal), and detection platform interface.

The maximum discharge standard of Cd^2+^ in industrial wastewater is 0.1 mg L^−1^ (0.9 μM).^8^ In the actual detection, as a real-time monitoring system, we hoped that the system would send an alarm signal before the concentration of Cd^2+^ in oily wastewater increases to the limit concentration rather than when it has reached the limit concentration. Therefore, we introduced a security coefficient (*k*) to reduce the concentration of Cd^2+^ and to trigger the alarm signal. After introducing the security coefficient *k*, the alarm concentration (*c*_limit_) can be obtained by eqn (2):

2 *c*_limit_ = *c*_standard_/*k*

where *c*_standard_ is 0.9 μM, and the value of *k* can be taken as 1.2. By calculation, the value of *c*_limit_ is 0.75 μM. The moving dimension of the gate voltage at the Dirac point (Δ*V*_dirac_) corresponding to the *c*_limit_ can be determined by eqn (3):3
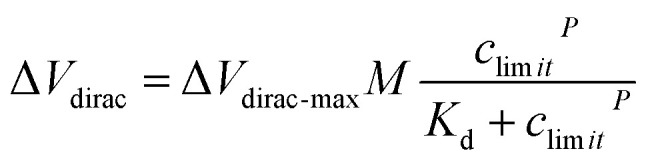
where *M* is the compensation factor, *c* is the concentration of Cd^2+^ in the solution, *P* is the synergistic factor, and *K*_d_ is the constant of dissociation equilibrium. The values of *M*, *K*_d_, and *P* could be obtained by the fitting process of eqn (1). In this study, the value of Δ*V*_dirac-max_ was 50 mV. After calculation, the Δ*V*_dirac_ that triggers the alarm signal was 46.8 mV. As shown in Fig. 4b, the circuit part did not send out an alarm signal when the graphene channel in the detection platform was exposed to solutions with Cd^2+^ concentrations of 10 pM, 100 pM, 1 nM, 10 nM, and 100 nM, respectively. When the graphene channel was exposed to Cd^2+^ solution with a concentration of 1 μM, in which the moving dimension of the gate voltage at the Dirac point exceeded 46.8 mV, the circuit board sent out a photoacoustic alarm signal.

### 3.4 Surface wettability tests of the oleophobic/hydrophilic membrane

**Fig. 5:**
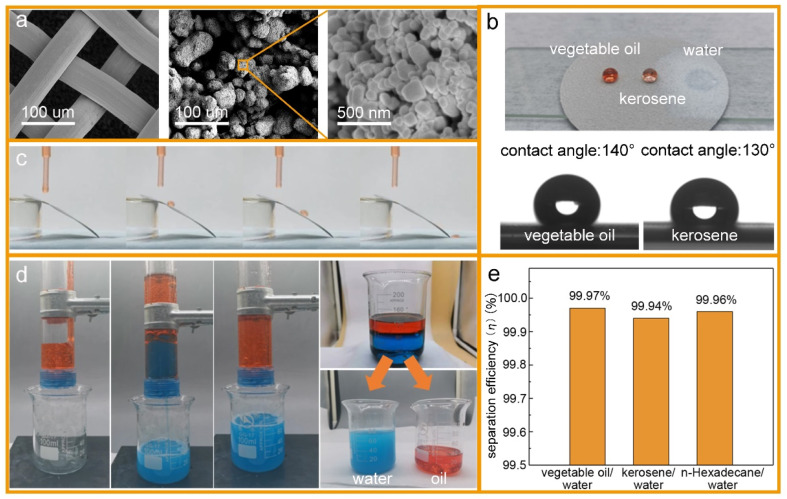
(a) SEM images of the surface of the untreated stainless-steel mesh and the stainless-steel mesh after treatment. The TiO_2_ nanoparticles induced multilevel roughness on the surface of the stainless-steel mesh. (b) Wetting characteristics of vegetable oil, kerosene, and water on the surface. The membrane surface showed hydrophilicity and hydrophobicity. (c) Oil droplet rolling test on the membrane surface. (d) Oil–water separation experiment (e) separation efficiency (*η*) of different types (vegetable oil/water, kerosene/water, *n*-hexadecane/water) of oil–water mixtures by the isolation membrane.

Surface wettability tests were performed after the preparation of the oleophobic/hydrophilic membrane. First, the surface topography of the membrane was observed by scanning electron microscopy (SEM) (Fig. 5a). The SEM images indicated that the TiO_2_ nanoparticles induced multilevel roughness on the surface of the stainless-steel mesh, which enhanced the surface wettability by increasing the surface area.^28^ The contact angles of vegetable oil, kerosene, and water on the membrane surface are shown in Fig. 5b. The member surface showed good hydrophobicity with a contact angle of 140° to vegetable oil and 130° to kerosene. By contrast, water could wet the membrane immediately. In addition, oil droplets could roll off the surface of the slanted membrane without any trace (Fig. 5c). For oil–water separation, three oil/water mixtures (vegetable oil/water, kerosene/water, and *n*-hexadecane/water) were prepared. Oils and water were dyed by Sudan III and methylene blue, respectively, before separation so that the oil–water boundary could be investigated clearly. In the process of the experiment, the water passed through the membrane while the oils were trapped on top of the membrane surface (Fig. 5d). After separation, the content of oil in the separated water was measured by an infrared spectrophotometric oil meter, and the oil–water separation efficiency was calculated by using eqn (4):4
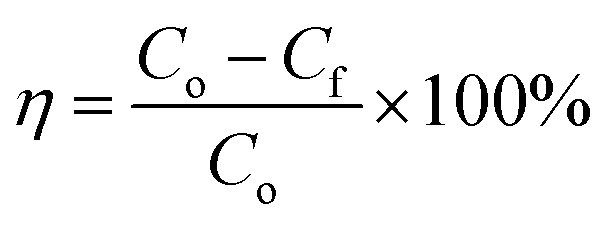
where *C*_o_ and *C*_f_ are the oil concentration before and after separation, respectively. The experimental steps for measuring *C*_f_ are summarized in the ESI.† By calculation, the *η* values of the membrane for vegetable oil/water, kerosene/water, and *n*-hexadecane/water three mixtures were 99.97%, 99.94%, 99.96%, respectively (Fig. 5e). These results show that the membrane exhibited good oleophobicity and hydrophilicity, and has high separation efficiency for oil/water mixtures.

### 3.5 Experiments to verify the effectiveness of the monitoring system

**Fig. 6:**
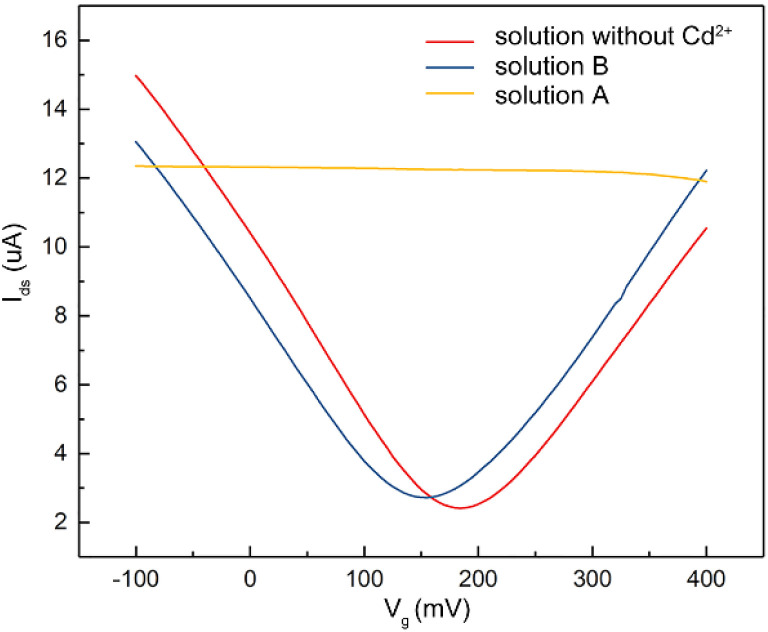
Transfer characteristic curves measured (*V*_ds_: 10 mV) during exposure of the A-GFET detecting platform to oily wastewater with a Cd^2+^ concentration of 100 nM before (solution A) and after (solution B) filtration by the isolation membrane, respectively, showing that the isolation membrane could avoid the influence of oil on the test results effectively.

To verify that the membrane could effectively eliminate the influence of oil pollution on the detection performance of the A-GFET detection platform for Cd^2+^ in oily wastewater, an oil-containing solution was prepared by adding 25 mL oil to 100 mL Cd^2+^ solution with a concentration of 100 nM and then stirred magnetically at room temperature for 30 min. Subsequently, the solution was divided equally into two parts labeled solution A and solution B. Solution A was directly added to the graphene channel in the A-GFET detecting platform, and solution B was added to the graphene channel after filtration by the membrane. The transfer characteristic curves of graphene's response to solution A and solution B are shown in Fig. 6. Compared with solution B, in which the transfer characteristic curve of graphene was shifted 35 mV to the left along the *x*-axis, the correct transfer characteristic curve of graphene could not be obtained under the influence of oil when the A-GFET detecting platform was exposed to solution A, indicating the membrane can avoid the influence of oil on the detection performance.

**Fig. 7:**
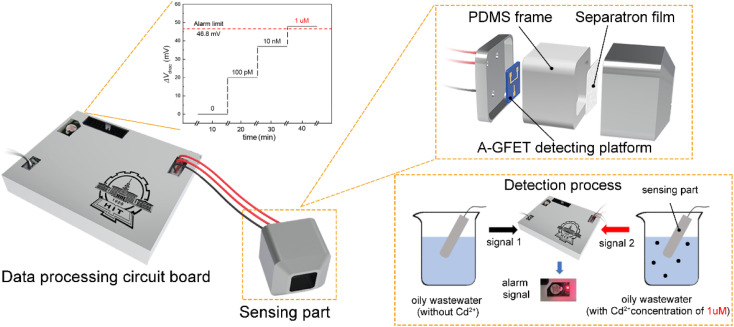
Real-time monitoring and alarm system for Cd^2+^ in oily wastewater. The system will send out a photoacoustic alarm signal when the concentration of Cd^2+^ in the monitored liquid environment increases abnormally to exceed the safe value.

After fabrication, the Cd^2+^-monitoring system was programmed to detect the transfer characteristic curve of graphene every 10 min. The voltage at the Dirac point in each detection was screened out to subtract it from the first detection, except for the first one. The Cd^2+^-monitoring system will send out a photoacoustic alarm signal until the deviations exceed the preset value. To investigate the monitoring ability of the system to Cd^2+^, the sensing part of the Cd^2+^ monitoring system was immersed in artificial oily wastewater without Cd^2+^. Then Cd^2+^ was added to the artificial oily wastewater every 10 min. The oil was blocked from the Cd^2+^-monitoring system by the barrier of the membrane, while the Cd^2+^ could pass through the membrane and contact with the graphene channel of the A-GFET detecting platform, thereby achieving the concentration monitoring of Cd^2+^. During detection, the concentration of Cd^2+^ in the oily wastewater was increased to 100 pM, 10 nM, and 1 μM by adding Cd^2+^ every 10 min. The monitoring system sent out a photoacoustic alarm signal until the Cd^2+^concentration went up to 1 μM with a preset value of 46.8 mV. Furthermore, the system did not send out an alarm signal when the sensing part was immersed in oily wastewater with a concentration of Cd^2+^ less than 1 μM (100 pM and 10 nM) and kept in solution for 30 min (Fig. 7).

## 4 Conclusions

In this work, a Cd^2+^-monitoring system for real-time monitoring of the Cd^2+^ concentration in oily wastewater, consisting of A-GFET detecting platform, oleophobic/hydrophilic membrane, and monitoring-alarm circuit, was presented. First, the A-GFET detecting platform as a key component of the monitoring system could respond to changes in the Cd^2+^ concentration within 10 min with a limit of detection (LOD) of 0.125 pM. Also, compared with control ions, such as Cr^3+^, Pb^2+^, Mg^2+^, and Fe^3+^, the detecting platform exhibited a high specificity to Cd^2+^. Second, the oleophobic/hydrophilic membrane was prepared using the fluorosurfactant FS-50, TiO_2_ nanoparticles, and stainless-steel mesh to stop the oil from contacting the graphene channel. The membrane exhibited a good oleophobic property with a contact angle of 140° to vegetable oil and 130° to kerosene. Also, the membrane also exhibited a high separation efficiency for oil/water mixtures. Finally, monitoring-alarm circuits, including power supply, voltage output, signal acquisition, signal processing, and photoacoustic alarm circuits, were designed to collect and process the signal produced by the A-GFET detecting platform. The collected signal was processed by the microcontroller STM32F429IGT6 after being converted into the voltage signal by an amplifier circuit. Following this, the instructions were sent to the alarm module if the alarm condition was triggered. The Cd^2+^-monitoring system could achieve Cd^2+^-concentration monitoring in oily wastewater with different limits of Cd^2+^ concentration by changing the alarm condition, thus demonstrating great potential for monitoring the concentration of heavy metal ions.

## Conflicts of interest

There are no conflicts to declare.

## Supplementary Material

NA-005-D2NA00416J-s001

## References

[cit1] Guo Y., Sun Y., Li Z., Feng S., Yang R., Qu L. (2022). J. Hazard. Mater..

[cit2] Song X., Zhang R., Wang Y., Feng M., Zhang H., Wang S., Cao J., Xie T. (2020). Talanta.

[cit3] Bilge S., Karadurmus L., Sınağ A., Ozkan S. A. (2021). TrAC, Trends Anal. Chem..

[cit4] Guo W., Zhang C., Ma T., Liu X., Chen Z., Li S., Deng Y. (2021). J. Nanobiotechnology.

[cit5] Lee Y. F., Huang C. C. (2011). ACS Appl. Mater. Interfaces.

[cit6] Liu C., Liang X., Liu J., Lei X., Zhao X. (2017). J. Colloid Interface Sci..

[cit7] Nidya M., Umadevi M., Rajkumar B. J. M. (2014). Spectrochim. Acta - A: Mol. Biomol..

[cit8] State Environmental Protection Administration of China , Integrated Wastewater Discharge Standard (GB 8978-1996), 1996

[cit9] Gasparik J., Vladarova D., Capcarova M., Smehyl P., Slamecka J., Garaj P., Stawarz R., Massanyi P. (2010). J. Environ. Sci. Health, Part A: Toxic/Hazard. Subst. Environ. Eng..

[cit10] Pohl P. (2009). TrAC, Trends Anal. Chem..

[cit11] Van Meel K., Smekens A., Behets M., Kazandjian P., Van Grieken R. (2007). Anal. Chem..

[cit12] Wang X., Shen C., Zhou C., Bu Y., Yan X. (2021). Chem. Eng. J..

[cit13] Novoselov K. S., Geim A. K., V Morozov S., Jiang D., Zhang Y., V Dubonos S., V Grigorieva I., Firsov A. A. (2004). Science.

[cit14] Kwong Hong Tsang D., Lieberthal T. J., Watts C., Dunlop I. E., Ramadan S., del Rio Hernandez A. E., Klein N. (2019). Sci. Rep..

[cit15] Ramadan S., Lobo R., Zhang Y., Xu L., Shaforost O., Tsang D. K. H., Feng J., Yin T., Qiao M., Rajeshirke A., Jiao L. R., Petrov P. K., Dunlop I. E., Titirici M. M., Klein N. (2021). ACS Appl. Mater. Interfaces.

[cit16] Ni S., Zhuo Z., Pan Y., Yu Y., Li F., Liu J., Wang L., Wu X., Li D., Wan Y., Zhang L., Yang Z., Zhang B. T., Lu A., Zhang G. (2021). ACS Appl. Mater. Interfaces.

[cit17] Röthlisberger P., Hollenstein M. (2018). Adv. Drug Deliv. Rev..

[cit18] Zhu G., Chen X. (2018). Adv. Drug Deliv. Rev..

[cit19] Ping J., Vishnubhotla R., Vrudhula A., Johnson A. T. C. (2016). ACS Nano.

[cit20] Hao Z., Wang Z., Li Y., Zhu Y., Wang X., De Moraes C. G., Pan Y., Zhao X., Lin Q. (2018). Nanoscale.

[cit21] Wang Z., Hao Z. (2022). Cell Rep. Phys. Sci..

[cit22] Liu S., Fu Y., Xiong C., Liu Z., Zheng L., Yan F. (2018). ACS Appl. Mater. Interfaces.

[cit23] An J. H., Park S. J., Kwon O. S., Bae J., Jang J. (2013). ACS Nano.

[cit24] Li Y., Wang C., Zhu Y., Zhou X., Xiang Y., He M., Zeng S. (2017). Biosens. Bioelectron..

[cit25] Wang R., Cao Y., Qu H., Wang Y., Zheng L. (2022). Talanta.

[cit26] Wang Z., Hao Z., Wang X., Huang C., Lin Q., Zhao X., Pan Y. (2021). Adv. Funct. Mater..

[cit27] Kota A. K., Kwon G., Choi W., Mabry J. M., Tuteja A. (2012). Nat. Commun..

[cit28] Li F., Wang Z., Huang S., Pan Y., Zhao X. (2018). Adv. Funct. Mater..

[cit29] Liu L., Pan Y., Jiang K., Zhao X. (2020). Appl. Surf. Sci..

[cit30] Yue X., Li Z., Zhang T., Yang D., Qiu F. (2019). Chem. Eng. J..

[cit31] Hao Z., Luo Y., Huang C., Wang Z., Song G., Pan Y., Zhao X., Liu S. (2021). Small.

