# Supplementary material for: Monitoring Cd2+ in oily wastewater using an aptamer-graphene field-effect transistor with a selective wetting surface
Source: Nanoscale Adv. 2022 Dec 13;5(5):1416–24. doi: 10.1039/d2na00416j (PMC9972544; doi:10.1039/d2na00416j)
Supplement: NA-005-D2NA00416J-s001 [file NA-005-D2NA00416J-s001.pdf]

Electronic Supplementary Material (ESI) for Nanoscale Advances.
This journal is © The Royal Society of Chemistry 2022

Supporting Information

Monitoring Cd^{2+} in oily wastewater using an aptamer-graphene field effect transistor with a selective wetting surface

Hao Wang[1], [2], Zhuang Hao[1], [2], Cong Huang[1], [2], Feiran Li* [1], [2], Yunlu Pan*[1], [2]
[1] Key Laboratory of Micro-systems and Micro-structures Manufacturing of Ministry of Education, Harbin Institute of Technology, Harbin 150001, Heilongjiang, China
[2] School of Mechatronics Engineering, Harbin Institute of Technology, Harbin 150001, Heilongjiang, China
*Corresponding author: yunlupan@hit.edu.cn (Yunlu Pan) and lifeiran@hit.edu.cn Feiran Li

1. The fabrication of graphene-based field effect transistor (GFET)

1.1 Au electrode fabrication

The fabrication of the Au electrode was shown in Fig.S1. Firstly, the photoresist LOR 3A was rotated onto the surface of the Si substrate (3500 r/min, 1min), and then the Si substrate was heated on a hot plate at 170℃ for 5min to solidify the photoresist. Secondly, the photoresist AZ1512 was rotated on LOR 3A similarly (4000r/min, 1min, heated at 110℃ for 2min). Then, the Si substrate was exposed for 7s by the lithography machine and subsequently immersed in the developer for 30s. The developer was washed with deionized water. To increase the adhesion between Au and substrate, 10nm of Cr was evaporated on the surface of the substrate before evaporating 30nm of Au. After deposition of Cr and Au, the substrate was finally immersed in Remover PG to remove the photoresists.

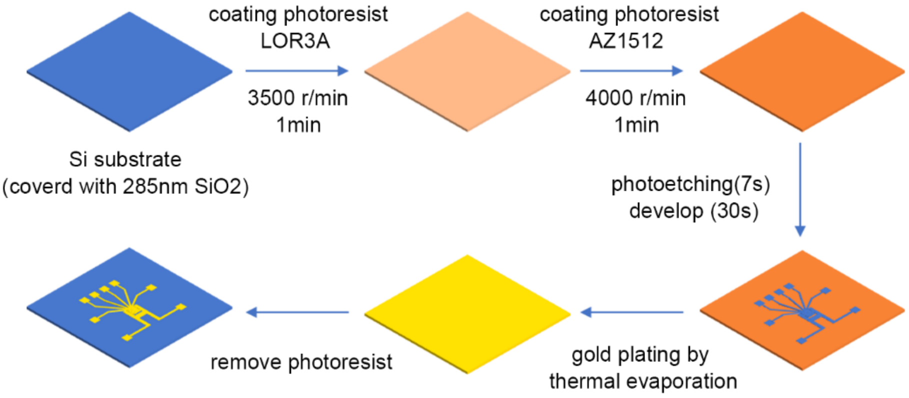

Fig.S1 Fabrication process of the Au electrode on Si substrate

1.2 Transfer process of graphene

Before transferring the graphene, 5% Ammonium persulfate (APS) solution was prepared to etch the copper substrate. Chemical vapor deposition (CVD) graphene grown on a copper substrate was cut into pieces of 3mm×3mm. The graphene pieces were floating on the APS solution for 3 hours, with copper contacting with APS. After etching Cu, the graphene pieces were transferred onto the surface of DI water by a glass slide and stayed for 30 min to wash APS. The graphene was finally transferred on the Au electrode to fabricate the graphene-based field effect transistor (GFET).

After transferring the graphene, the GFET was stored overnight in a vacuum chamber to make the graphene stick to the Si substrate. After that, the GFET was heated at 180℃ for an hour to remove the internal stress. Finally, the GFET was immersed in acetone to remove PMMA, which covered on the graphene as a protective layer, and then was washed with isopropanol and DI water successively.

2. Detecting principle of A-GFET platform

In this study, PASE was used as a binding molecule to achieve the immobilization of the Cd^{2+} aptamer on the surface of graphene. As shown in Fig.S2, the pyrenyl group at one end of the PASE molecular was combined with graphene by π-π stacking, ester group at the other end was combined with the amino group on aptamer by Schiff Base Condensation to achieve modification of the aptamer on the graphene surface. During

detection, the aptamers transform from an extended long-chain structure into a compact and stable structure after combing with Cd^{2+}, which can pull more negative charges produced by the hydrolysis of phosphate groups in aptamers close to the surface of the graphene (Fig.S3). This change introduces N-type doping to graphene, resulting in a left shift of the graphene transfer characteristic curves along the X-axis. And the curves shift to the left continually as the Cd^{2+} concentrations increase within the range of a certain density.

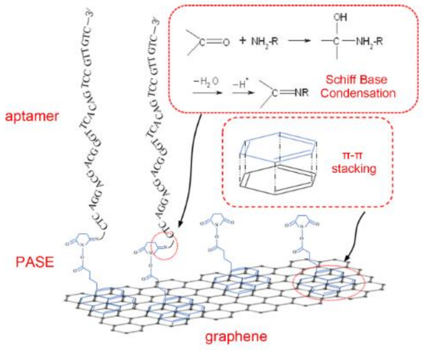

Fig.S2 The principle of modifying PASE and aptamer on graphene surface

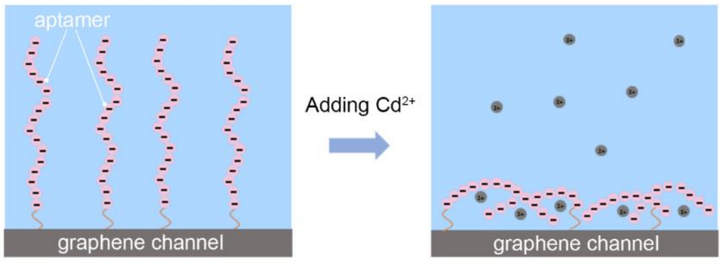

Fig.S3 Detecting principle of A-GFET for Cd^{2+}

3. Calculation of the limit of detection (LOD)

As shown in Fig.3d, the normalized data can be fitted by the function (1):

$$y = \frac{\Delta V_{dirac}}{\Delta V_{dirac\_max}} = M\frac{c^P}{K_d + c^P}$$

Taking concentration (c) as the dependent variable and signal $\Delta V_{dirac} / \Delta V_{dirac\_max}$ (y) as the independent variable, the inverse function of function (1) can be obtained:

$$\mathrm{c} = \left(\frac{K_d y}{M - y}\right)^{\frac{1}{P}} \tag{5}$$

According to the 3σ principle, the response signal of the sensor at the limit concentration can be expressed as: $y=3\sigma$, where σ is the standard deviation between the actual signal and the fitted value, and can be calculated by the function (6):

$$\sigma = \sqrt{\frac{\sum_{i=1}^{n}\left(y_i - \overline{y_i}\right)^2}{n}} \tag{6}$$

Where y_i is the actual value, $\overline{y_i}$ is the fitted value.

In conclusion, the limit of detection (c_{LOD}) can be obtained by the be function (7):

$$c_{LOD} = (\frac{3K_d\sigma}{M-\sigma})^{\frac{1}{P}} \tag{7}$$

By calculation, the c_{LOD} is 0.125pM.

4. The physical model of the oleophobic/hydrophilic membrane

As shown in Fig.S4, the oleophobic/hydrophilic membrane is based on stainless steel mesh covered with TiO_2 nanoparticles and fluorosurfactant FS-50. The fluorocarbon chain in the structure of FS-50 can reduce the surface energy, making the surface appear to be oleophobic. While the polar group can increase the polar component of the surface energy, making the surface appear to be hydrophilic. TiO_2 nanoparticles attached to the surface of stainless-steel mesh can increase the surface roughness, enhancing the selective wettability of the surface. Contact Angle of water and oil on stainless steel mesh, stainless steel mesh sprayed with FS-50, stainless steel mesh sprayed with FS-50 and TiO_2 nanoparticles are shown in Fig.S5 and Fig.S6 respectively.

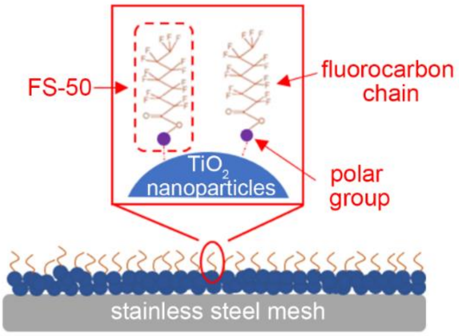

Fig.S4 The physical model of the oleophobic/hydrophilic membrane

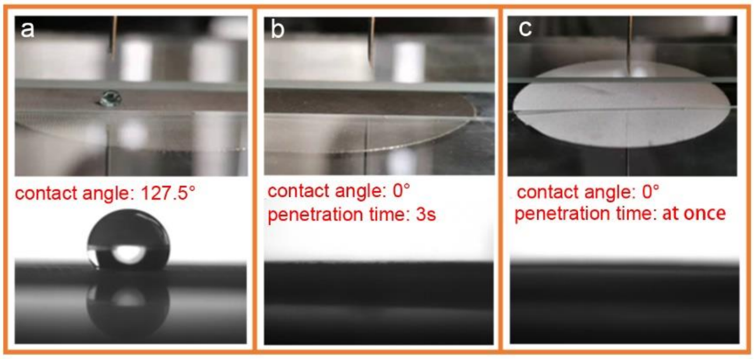

Fig.S5 Contact Angle (CA) of water on stainless steel mesh(a), stainless steel mesh sprayed with FS-50(b), stainless steel mesh sprayed with FS-50 and TiO_2 nanoparticles(c)

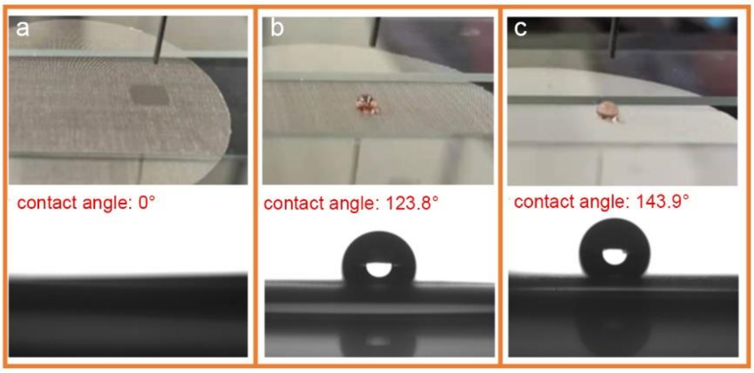

Fig.S6 Contact Angle (CA) of oil on stainless steel mesh(a), stainless steel mesh sprayed with FS-50(b), stainless steel mesh sprayed with FS-50 and TiO_2 nanoparticles(c)

5. The composition of monitoring system and alarm experiment

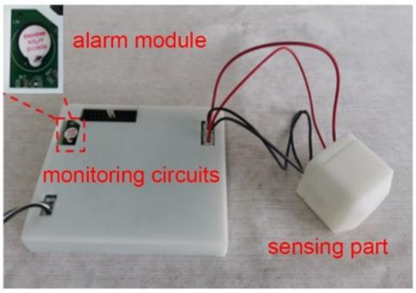

Fig.S7 The composition of monitoring system

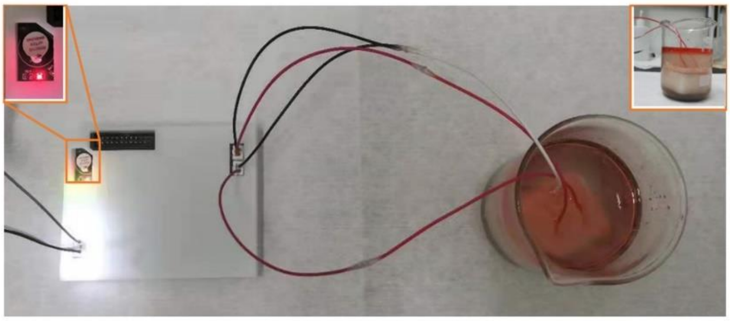

Fig.S8 Alarm experiment

To prepare oily wastewater, firstly, water samples without cadmium ions were obtained by mixing deionized water with soil. Secondly, 10mL oil was added to 90mL water sample to obtain artificial oily wastewater. The Cd^{2+} concentration is controlled by adding cadmium chloride ($CdCl_2$) to the artificial oily wastewater.

6. IV transfer characteristics of the experiment of selectivity

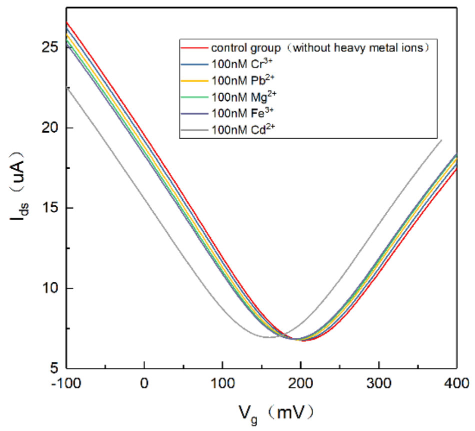

Fig.S9 IV transfer characteristic curves of the response signal of GFET detecting platform to Cr^{3+}, Pb^{2+}, Mg^{2+}, Fe^{3+} and Cd^{2+}

In the figure of the IV transfer characteristic curves, the gate voltage (Vg) of Dirac point changes very little (within 13mV) when the four control ions were detected. In contrast, the Vg of Dirac point was reduced by 45mV when the detecting platform exposure to Cd^{2+} solution. The signal response of this detecting platform to Cd^{2+} is more than three times than that of the control ions, indicating that this detecting platform has good selectivity for Cd^{2+}.

7. **Emission standards of some heavy metals**

Table S1 The maximum allowable emission concentration of some heavy metals in wastewater

mg/L

Number	Contaminants	Maximum allowable emission concentration
1	Hg	0.05
2	Cd	0.1
3	Cr	1.5
4	As	0.5
5	Pb	1.0
6	Ni	1.0
7	Be	0.005
8	Ag	0.5

8. **Measurement of the oil concentration in water after oil-water separation**

Before measurement, the oil in water after separation is first extracted with tetrachloroethylene. This operation is done by a jet extractor. The specific experimental steps are as follows:

(1) Add 10ml water sample and 50ml tetrachloroethylene to the extraction bottle;

(2) Set the extraction time to 2 minutes. Then click the “Start” button and wait for the end of the extraction process.

(3) Place the glass wool in the glass funnel. Take an appropriate amount of anhydrous sodium sulfate and spread it on the glass wool.

(4) Open the knob under the extraction bottle. Collect tetrachloroethylene at the bottom of the extraction bottle into the volumetric flask through the funnel containing

anhydrous sodium sulfate.

During measurement, firstly, we need to input the relevant measurement parameters. For example, the volume of the water sample is 10ml, the volume of the extracting solution is 50 ml. Secondly, a blank spectrogram is measured by putting tetrachloroethylene into the cuvette. Finally, we pour the solution in the volumetric flask into the cuvette for measurement to obtain the concentration of the oil in the water after the separation.

9. **The explanation of different doping of graphene by PASE and aptamer**

Before detecting Cd^{2+}, The transfer characteristic curves of graphene after PASE and aptamer modification was firstly measured. The results show that transfer curve of graphene shifts to higher gate potentials while immobilizing PASE and lower gate potentials while grafting the aptamer.

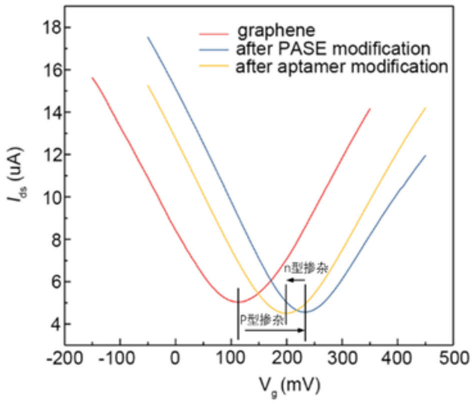

Fig.10 Electrical characterization of graphene, graphene after modifying PASE and graphene after modifying aptamer.

This experimental phenomenon can be explained as:

The molecular structure of PASE includes a hexagonal pyrene group and a N-hydroxysuccinimide group. The positive and negative charge centers of PASE molecular do not coincide. The negative charge is concentrated on the pyrene group while the positive charge is concentrated on the N-hydroxysuccinimide group (Hao et al., 2020). Since the ability of the N-hydroxysuccinimide group to attract electrons is greater than that of the pyrene group to repel electrons, the electrons will be transferred from graphene to PASE when the PASE molecules are modified on the surface of graphene by π-π stacking. This change will lead to a decrease in the number of free electrons in graphene (the number of holes increases). This results in P-type doping of graphene, causing the transfer characteristic curve of graphene to shifts to higher gate potentials.

In the liquid environment, the phosphate groups in the aptamer will hydrolyze and become negatively charged (Stotzky, 2000). When the aptamer is modified on graphene, the same amount of positive charges will accumulate on the surface of graphene under the action of electrostatic induction. This change will lead to an increase in the number of free electrons in graphene according to the conservation of charge. This results in n-type doping of graphene, causing the transfer characteristic curve of graphene to shifts to lower gate potentials.

In the process of detecting Cd^{2+}, the aptamers transform from an extended long-chain structure into a compact and stable structure after combing with Cd^{2+}, which can pull more negative charges produced by the hydrolysis of phosphate groups in aptamers close to the surface of the graphene. This change further increases the number of free electrons in graphene, causing N-type doping in graphene. Therefore, the transfer characteristic curve of graphene in the detecting process shifts to lower gate potentials.

10. **The comparison of some important characteristics of the proposed detecting method to Cd^{2+} with the previously reported studies.**

Table S2 Comparison of the proposed detecting method to Cd^{2+} with the previously reported studies.

Method[a]	Sensing material[b]	LOD (nM)	Response time (min)	Sensitivity	reference
SPR	pHEMA	2.10×10^{-1}	6-7	—	(Bakhshpour and Denizli, 2020)
DPASV	Cd–IIP	3.13	5	0.92uA/ppb	(Ghanei-Motlagh and Taher, 2017)
Voltammetry	MPA-GSH	5	10	—	(Chow et al., 2005)
CR	IIP/rGO	17.3	—	$0.14ppb^{-1}$	(Hu et al., 2021)
DPASV	APT-GCN	0.337	—	0.083VuA/dec	(Wang et al., 2018)
optical detection	MoS_2@TMPyP	72	20	—	(Yin et al., 2017)
SWASV	HP-β-CD-RGO/Nafion/GCE	6.73×10^{-2}	2	0.281uA/nM	(Lv et al., 2013)
TCC	APT-GO	1.25×10^{-4}	8	$7.643\times10^{-2}nM^{-1}$	this work

a: SPR: surface plasmon resonance; DPASV: differential anodic stripping voltammetry; CR: chemiresistor; SWASV: square wave anodic stripping voltammetry; TCC: transfer characteristic curve

b: pHEMA: Poly(hydroxyethyl methacrylate); Cd–IIP: nanostructured cadmium ion imprinted polymer; MPA-GSH: 3-mercaptopropionic acid-glutathione; IIP/rGO: ion imprinted polymer-functionalized reduced graphene oxide; APT-GCN: aptamer-graphene oxide/graphitic carbon nitride; MoS2@TMPyP： a nanocomposite consisted of MoS2 nanosheets and 5,10,15,20-tetrakis(1-methyl-4-pyridinio)porphyrintetra(p-toluenesulfonate); HP-β-CD-RGO/Nafion/GCE: hydroxypropyl-β-cyclo-dextrin decorated reduced GO/Nafion/glassy carbon electrode; APT-GO: aptamer-graphene

Reference

Li, F., Wang, Z., Huang, S., Pan, Y., Zhao, X., 2018. Flexible, Durable, and Unconditioned Superoleophobic/Superhydrophilic Surfaces for Controllable Transport and Oil–Water Separation. Adv. Funct. Mater. 28. https://doi.org/10.1002/adfm.201706867

Hao, Z., Luo, Y., Huang, C., Wang, Z., Song, G., Pan, Y., Zhao, X., Liu, S., 2021. An Intelligent Graphene-Based Biosensing Device for Cytokine Storm Syndrome Biomarkers Detection in Human Biofluids. Small 17. https://doi.org/10.1002/smll.202101508

Hao, Z., Pan, Y., Huang, C., Wang, Z., Lin, Q., Zhao, X., Liu, S., 2020. Modulating the Linker Immobilization Density on Aptameric Graphene Field Effect Transistors Using an Electric Field. ACS Sensors 5, 2503–2513. https://doi.org/10.1021/acssensors.0c00752

Stotzky, G., 2000. Persistence and Biological Activity in Soil of Insecticidal Proteins from Bacillus thuringiensis and of Bacterial DNA Bound on Clays and Humic Acids. J. Environ. Qual. 29, 691–705. https://doi.org/10.2134/jeq2000.00472425002900030003x

Bakhshpour, M., Denizli, A., 2020. Highly sensitive detection of Cd(II) ions using ion-imprinted surface plasmon resonance sensors. Microchem. J. 159, 105572. https://doi.org/10.1016/j.microc.2020.105572

Chow, E., Hibbert, D.B., Gooding, J.J., 2005. Voltammetric detection of cadmium ions at glutathione-modified gold electrodes. Analyst 130, 831–837. https://doi.org/10.1039/b416831c

Ghanei-Motlagh, M., Taher, M.A., 2017. Novel imprinted polymeric nanoparticles prepared by sol–gel technique for electrochemical detection of toxic cadmium(II) ions. Chem. Eng. J. 327, 135–141. https://doi.org/10.1016/j.cej.2017.06.091

Hu, J., Sedki, M., Shen, Y., Mulchandani, A., Gao, G., 2021. Chemiresistor sensor based on ion-imprinted polymer (IIP)-functionalized rGO for Cd(II) ions in water. Sensors Actuators, B Chem. 346, 130474. https://doi.org/10.1016/j.snb.2021.130474

Lv, M., Wang, X., Li, J., Yang, X., Zhang, C., Yang, J., Hu, H., 2013. Cyclodextrin-reduced graphene oxide hybrid nanosheets for the simultaneous determination of lead(II) and cadmium(II) using square wave anodic stripping voltammetry. Electrochim. Acta 108, 412–420. https://doi.org/10.1016/j.electacta.2013.06.099

Wang, X., Gao, W., Yan, W., Li, P., Zou, H., Wei, Z., Guan, W., Ma, Y., Wu, S., Yu, Y., Ding, K., 2018. A Novel Aptasensor Based on Graphene/Graphite Carbon Nitride Nanocomposites for Cadmium Detection with High Selectivity and Sensitivity. ACS Appl. Nano Mater. 1, 2341–2346. https://doi.org/10.1021/acsanm.8b00380

Yin, W., Dong, X., Yu, J., Pan, J., Yao, Z., Gu, Z., Zhao, Y., 2017. MoS2-Nanosheet-Assisted Coordination of Metal Ions with Porphyrin for Rapid Detection and Removal of Cadmium Ions in Aqueous Media. ACS Appl. Mater. Interfaces 9, 21362–21370. https://doi.org/10.1021/acsami.7b04185
